# Discovery of data quality issues in electronic health records: profound consequences for critical care medicine applications – a systematized review

**DOI:** 10.1186/s13054-025-05677-0

**Published:** 2026-01-08

**Authors:** João Brainer Clares de Andrade, Marconny Alexandre Oliveira de Medeiros Cavalcante, Thiago Luís Marques Lopes, João Marcos Secundino Treigher, Mateus Dutra Balsells, Júlia Lima Vasconcelos, Lis Cavalcante Monteiro, Déborah Danna da Silveira Mota

**Affiliations:** 1 Department of Health Informatics, Federal University of São Paulo (UNIFESP), São Paulo, Brazil; 2 Hospital Israelita Albert Einstein, São Paulo, Brazil; 3 School of Medicine, State University of Ceará (UECE), Fortaleza, Brazil; 4 Academic Research Organization Hospital Israelita Albert Einstein, Av. Albert Einstein, 627/701, São Paulo, ZIP 05652-900 SP – Brazil

**Keywords:** Electronic health records, Critical care, Digital health

## Abstract

**Introduction:**

Electronic health records (EHRs) in critical care settings generate vast amounts of data that increasingly drive machine learning (ML) models for clinical decision support, yet data quality issues may have profound consequences for downstream prediction, classification, and optimization applications. This study aims to systematically examine EHR data quality issues in critical care medicine and their impact on ML model performance, clinical outcomes, and patient safety.

**Methods:**

We conducted a systematized review following expert-based questions, searching MEDLINE, Embase, IEEE Xplore, ACM Digital Library, CINAHL, Google Scholar, DBLP, Web of Science, and the Cochrane Library. Six distinct questions addressed missing data patterns, temporal data quality, bias and health equity, multi-modal integration, real-time monitoring, and institutional variability.

**Results:**

281 relevant studies examining EHR data quality in critical care settings. After applying the eligibility criteria, 29 studies were selected. EHR data quality issues in critical care were pervasive and multifaceted. Missing data rates exceeded 80% for some variables, with 40% of predictive features being missingness indicators rather than actual values. EHR-related medication errors comprised 34% of all medication errors in ICUs, with one-third having life-threatening potential. Copy-paste prevalence reached 82% in residents’ progress notes. ML model performance degraded significantly under real-world conditions, with external validation showing AUC drops from 0.76 to 0.63 for sepsis detection models. Temporal data quality deteriorated throughout ICU stays, with vital sign quality degrading at 60–75% of average length of stay.

**Conclusion:**

Data quality issues in critical care EHRs create cascading effects that compromise ML model reliability, clinical decision-making, and patient safety. The evidence demonstrates an urgent need for systematic data quality monitoring, bias-aware assessment methods, and comprehensive quality improvement frameworks specifically designed for critical care environments.

**Supplementary Information:**

The online version contains supplementary material available at 10.1186/s13054-025-05677-0.

## Introduction

Critical care medicine has embraced the promise of artificial intelligence and machine learning to enhance clinical decision-making, predict patient deterioration, and optimize resource allocation [1]. However, the foundation of these advanced technologies — electronic health record (EHR) data — frequently suffers from quality issues that can have profound consequences for downstream applications [2]. The high-stakes, time-sensitive nature of critical care amplifies the potential impact of data quality problems, making this domain particularly vulnerable to the cascading effects of poor data integrity [3].

Recent advances in machine learning have enabled sophisticated predictive models for mortality risk, sepsis detection, and clinical deterioration warning systems [4]. Yet emerging evidence suggests that data quality issues in critical care EHRs may systematically undermine these applications, leading to biased predictions, reduced model generalizability, and potentially harmful clinical decisions [5]. The unique characteristics of intensive care environments — including high-frequency physiological monitoring, complex multi-system integration, and urgent clinical decision-making — create distinctive data quality challenges that require specialized understanding and targeted solutions [3].

This systematized review aims to comprehensively examine data quality issues in critical care EHRs and their impact on machine learning applications, clinical outcomes, and patient safety. Specifically, we seek to characterize the types and prevalence of data quality issues in critical care EHR systems, to quantify the impact of these issues on ML model performance and clinical decision support systems, to analyze the consequences for patient outcomes and healthcare equity; to evaluate methods for detecting and mitigating data quality problems and to identify best practices and future directions for improving data quality in critical care settings.

## Methods

### Study design and framework

This study employed a systematized review methodology to comprehensively examine EHR data quality issues in critical care settings and their impact on machine learning applications. A systematized review incorporates some elements of systematic review methodology while maintaining greater flexibility in approach and scope, making it particularly suitable for exploring complex, multidimensional topics where rigid systematic review protocols may be unnecessarily restrictive.

## Expert-derived research questions

Rather than employing traditional PICO frameworks, this review was guided by six expert-derived research questions developed through consultation with clinical informatics specialists, critical care physicians, and machine learning researchers. These questions were formulated to address the most pressing gaps identified by domain experts:

RQ1: How do missing data patterns in ICU environments affect the performance and reliability of predictive machine learning models, and what advanced imputation strategies show the greatest promise for maintaining model accuracy?

RQ2: What temporal data quality challenges emerge in longitudinal critical care episodes, and how do these impact time-series modeling approaches compared to cross-sectional analyses?

RQ3: How do EHR data quality issues contribute to health equity concerns and algorithmic bias in critical care ML applications, particularly across diverse patient demographics?

RQ4: What frameworks exist for assessing data quality in multi-modal ICU environments (physiological monitoring, laboratory results, imaging, clinical notes), and how does integration quality affect clinical decision support systems?

RQ5: How can real-time data quality monitoring be implemented in ICU settings to improve workflow efficiency and clinical outcomes compared to retrospective quality assessment approaches?

RQ6: What strategies address institutional variability in EHR data quality across multi-center critical care networks, and how does standardization impact cross-institutional model generalizability?

### Search strategy and information sources

We conducted searches across multiple databases selected for their relevance to the intersection of health informatics, critical care, and machine learning. Primary databases included PubMed for comprehensive medical literature coverage, IEEE Xplore for technical and engineering perspectives, ACM Digital Library for computer science and informatics contributions, and Google Scholar, DBLP and Web of Science for multidisciplinary coverage. Supplementary databases comprised Embase for biomedical literature with European focus, CINAHL for nursing and allied health perspectives, and the Cochrane Library for systematic reviews and controlled trials.

The search strategy employed a four-concept approach using Boolean operators, with terms iteratively refined based on preliminary searches and expert consultation. The first concept encompassed electronic health records using terms such as “electronic health record*”, “EHR”, “electronic medical record*”, “EMR”, “clinical information system*”, and “hospital information system*”. Data quality dimensions formed the second concept, incorporating “data quality”, “data integrity”, “data completeness”, “missing data”, “data accuracy”, “data timeliness”, “temporal shift*”, “data bias*”, and “data heterogeneity”. The critical care context was captured through terms including “critical care”, “intensive care”, “ICU”, “CICU”, “MICU”, “SICU”, “emergency department”, “acute care”, and “critical illness”. Machine learning applications comprised the fourth concept with terms such as “machine learning”, “artificial intelligence”, “predictive model*”, “clinical decision support”, “deep learning”, “neural network*”, and “algorithm*”. Search strings were systematically adapted for each database’s specific syntax and controlled vocabulary, including MeSH terms and EMTREE descriptors where applicable. The search strategies, adapted to each database, are included in the Supplementary Material.

### Eligibility criteria

Studies were included if they involved adult patients aged 18 years or older in critical care settings and examined EHR data quality assessment, measurement, or improvement interventions within critical care, intensive care, or emergency department environments. We included studies that reported on impacts on machine learning model performance, clinical decision support systems, or care quality outcomes. Eligible study designs encompassed primary research studies, systematic reviews, and technical reports published as peer-reviewed articles or conference proceedings from major venues including IEEE, ACM, and AMIA conferences. We restricted inclusion to publications from January 2010 to December 2024 to capture the modern EHR and machine learning era; no language restrictions were applied.

Studies were excluded if they focused exclusively on pediatric populations or outpatient settings, examined only EHR implementation or adoption without data quality assessment, or were published as conference abstracts without full text, editorials, or commentaries. We also excluded case reports with fewer than 10 participants, purely theoretical papers without empirical components, and studies with insufficient methodological detail for adequate assessment.

### Study selection and screening process

The study selection process was conducted in two phases by a single reviewer, acknowledging that systematized review methodology allows for single-reviewer approaches while maintaining methodological integrity. To ensure selection consistency, a random 10% subset of studies was double-screened by a second reviewer. During the initial phase, title and abstract screening was performed using a liberal inclusion approach to minimize the risk of missing relevant studies, with uncertain cases advanced to full-text review. The second phase involved detailed evaluation of full-text articles against the complete eligibility criteria, with systematic documentation of exclusion reasons for rejected studies. When uncertainties arose during the selection process, expert consultation was sought to ensure appropriate inclusion decisions.

### Data extraction strategy

Data extraction focused systematically on methodological characteristics and key findings relevant to the expert-derived research questions. For each included study, we extracted comprehensive study characteristics including study design and setting, sample size and patient population demographics, EHR system characteristics and data types examined, and machine learning approaches employed. We systematically captured data quality measures encompassing the types of data quality issues identified, measurement approaches and metrics utilized, temporal patterns and trends observed, and methods used for impact quantification. Key outcomes and findings were extracted including machine learning model performance impacts, effects on clinical outcomes, implementation challenges and solutions identified, and specific recommendations for practice provided by study authors.

### Quality assessment approach

Given the systematized review methodology and the diverse study designs anticipated in this emerging field, we employed a pragmatic quality assessment approach rather than formal risk of bias tools. This approach evaluated methodological adequacy by assessing the appropriateness of study design for addressing the stated research questions, reporting completeness by evaluating whether sufficient detail was provided for reproducibility, clinical relevance by assessing applicability to real-world critical care settings, and evidence strength by considering factors such as sample sizes, follow-up periods, and outcome measures used. Studies were not excluded based solely on quality concerns, recognizing the evolving nature of this research area, but quality assessments were used to inform the strength of conclusions drawn from the synthesized evidence.

### Data synthesis and analysis

Where appropriate, quantitative synthesis employed descriptive statistics to summarize the frequency and types of data quality issues identified across studies, common patterns in missing data rates, and the distribution of study characteristics and settings. A comprehensive narrative synthesis approach was implemented to organize findings according to the six expert-derived research questions, systematically identify recurring themes and patterns across studies, highlight contradictory findings and knowledge gaps, and develop evidence-based recommendations for future research and practice.

Based on the synthesized evidence, we developed a comprehensive taxonomy of EHR data quality issues specific to critical care environments, evidence-based recommendations for data quality assessment and improvement strategies, and practical guidelines for machine learning model development in the presence of data quality challenges. This framework development process integrated findings across all included studies to provide actionable guidance for researchers and practitioners working at the intersection of critical care informatics and machine learning.

### Limitations and methodological considerations

We acknowledge some limitations inherent to the systematized review approach:

- Single reviewer selection: While a subset was double-screened, the primary selection was conducted by one reviewer.
- Database scope: focus on major databases May have missed grey literature or specialized publications
- Rapid evolution: The fast-moving nature of ML and health informatics may mean recent developments are underrepresented.

These limitations were balanced against the advantages of the systematized approach, including greater flexibility to address complex, multidisciplinary questions and the ability to incorporate expert insights throughout the process.

### Reporting standards

This systematized review was conducted and reported in accordance with available guidance for evidence synthesis, incorporating applicable elements from PRISMA guidelines while acknowledging that formal PRISMA compliance is not required for systematized reviews.

## Results

### Overview of data quality issues

**Fig. 1:**
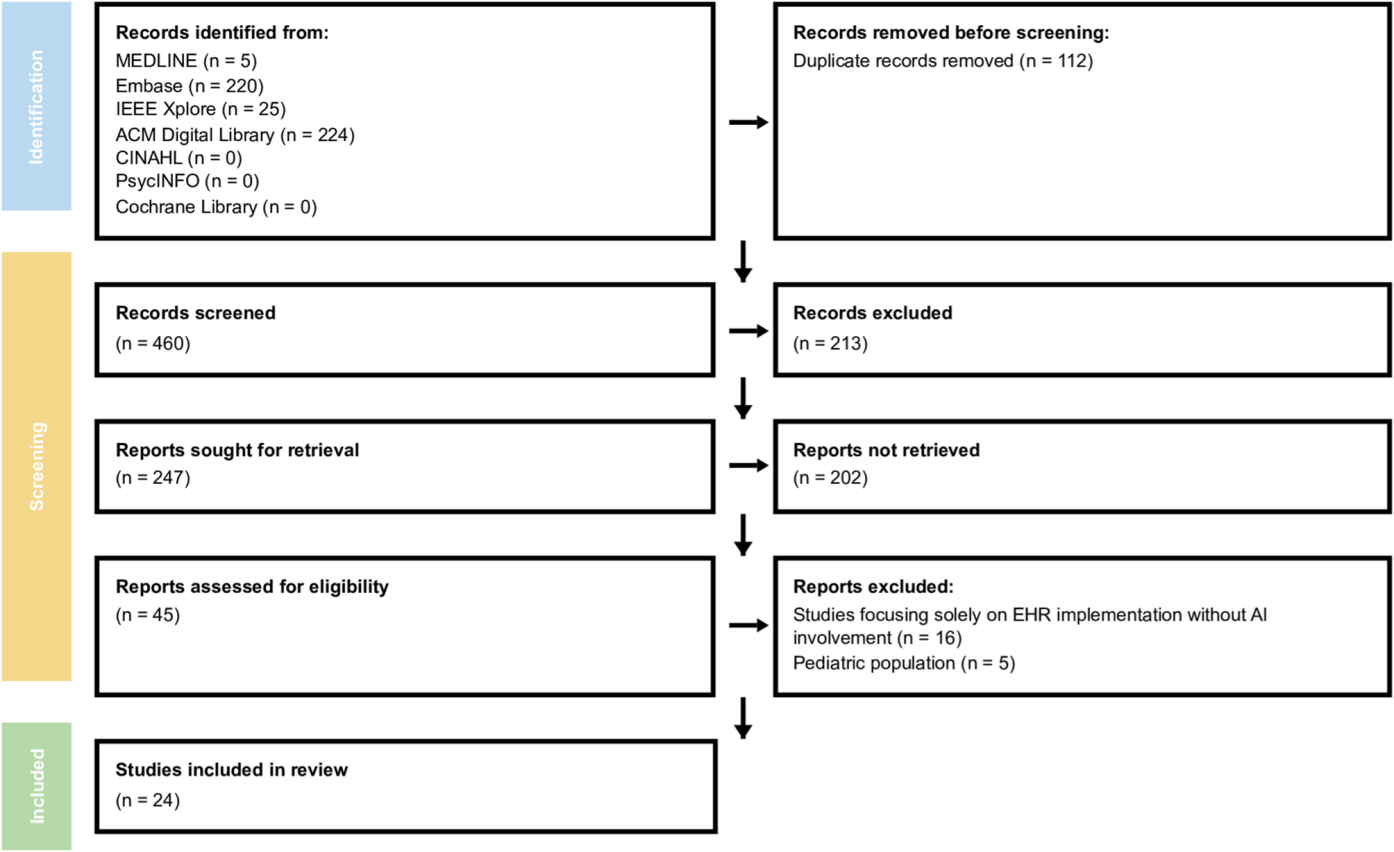
PRISMA flow diagram for systematized review

Our systematic review identified 281 relevant studies examining EHR data quality in critical care settings. After applying the eligibility criteria, in accordance with the PRISMA guidelines [4], 29 studies were selected [1, 3, 6–32] (Fig. 1). The evidence reveals pervasive and multifaceted data quality challenges that significantly impact clinical care and research applications [6]. General characteristics of the included studies are summarized in Table 1.

**Table 1:** General aspects of included studies

Study (Citation)	Clinical task	Data quality issue investigated	Method of comparison	Main results	Country
Jalilian et al. (2022) [1]	ICU clinical‑dashboard/data‑visualisation support	EHR information overload and poor usability degrading data quality	Narrative synthesis of usability studies and heuristic principles—no quantitative head‑to‑head test	Highlights redundant screens, data lag and high cognitive workload; proposes user‑centred dashboards to mitigate safety risks	United States
Johnson et al. (2023) [3]	Creation of critical‑care research dataset (MIMIC‑IV)	De‑identification, completeness & modular organisation for research use	Described acquisition‑transform‑de‑ID pipeline for BIDMC 2008‑2019 data	Released publicly‑available ICU/ED dataset spanning 2008‑2019, enabling reproducible research worldwide	United States
Lewis et al. (2023) [6]	Survey of EHR data‑quality assessment research	Completeness, correctness, concordance, plausibility, currency, conformance, bias	Systematic review of 103 papers (2013‑2023) by DQ dimension & method	Publications surged; completeness most assessed, yet no standardised DQA approach has emerged	United States
Rajkomar et al. (2018) [7]	Multi‑task EHR predictions (mortality, readmission, LOS, diagnoses)	Cross‑site heterogeneity without manual data harmonisation	Deep‑learning on raw FHIR data vs. traditional models at two hospitals	DL achieved AUROC 0.93–0.94 for mortality and beat all baselines on every task	United States
Kim et al. (2024) [8]	Guidance for EHR‑based cohort studies	Missing data, heterogeneity & systematic bias in EHRs	Narrative review outlining error sources and mitigation strategies	Identifies missing data as dominant bias; recommends proactive validation and bias‑mitigation framework	United States
Sharafoddini et al. (2019) [9]	ICU mortality prediction using lab data	Informative missingness in EHR laboratory tests	AUROC of models built only on “missingness indicators” vs. models using observed values ± indicators	Missingness indicators alone yielded AUROC ≈ 0.68; adding them boosted performance by ≤ 0.043	Canada
Getzen et al. (2023) [10]	Disease prediction using medical knowledge graphs	Missing data simulation & data fragmentation	Knowledge-graph-based missingness vs. random removal impact on ML models	Missing data reduced AUC, especially in underserved groups; knowledge-graph removal had greater impact	United States
Xie et al. (2022) [11]	Review of temporal‑data deep‑learning for clinical prediction	Irregularity, sparsity, heterogeneity in temporal EHR	Systematic review analyzing 98 DL papers by challenge/method	Identified four key challenges; RNN/LSTM most common; need integrated, interpretable solutions	Singapore
Goodwin et al. (2022) [12]	Accurate temporal data for critical‑care analytics	Timing errors & temporal uncertainty from unsynchronised devices	Narrative review comparing documented clock drift/error sources with best‑practice master‑clock synchronisation	Finds timekeeping errors pervasive; recommends master‑clock synchronisation and explicit modelling of temporal uncertainty	Canada
Afshar et al. (2021) [13]	High‑Freq ICU Vital‑Signs Capture	Completeness/Accuracy/Timeliness of minute-by-minute vital signs	Descriptive analysis of HR, RR, SpO₂, and ABP streams in MIMIC-III	~ 30% of cases had less than 1 min of data; only 12.5% had ≥ 99% HR coverage	United States
Gianfrancesco et al. (2018) [14]	Bias appraisal for ML‑based clinical decision support	Missing data, sample imbalance & misclassification in EHR datasets	Conceptual review contrasting bias mechanisms and mitigation tactics	Warns that unchecked data‑set bias can amplify health inequities and offers practical safeguards	United States
Gonzalez et al. (2024) [15]	24‑h AI decision‑support for ICU patient stratification	Data heterogeneity, system integration & model transparency	Thematic review of published ICU ML algorithms and deployment challenges	Notes sepsis‑prediction AUROC > 0.90 in several studies but stresses interoperability, ethics and bias as barriers to bedside use	Portugal
Bowman et al. (2013) [16]	EHR Information Integrity	EHR‑Induced Documentation Errors	Narrative review of unintended consequences	Flawed design and usability issues result in safety-threatening errors, highlighting the need for standardized design practices	United States
Ni et al. (2020) [17]	Medication‑administration error detection (NICU)	Linkage/concordance between smart‑pump records and EHR MARs	Algorithm aligned SPRs with orders; compared discrepancies caught by SPR vs. MAR	70% SPRs linkable; SPRs flagged 682 dosing discrepancies vs. 321 in MAR with minimal overlap, showing added value	United States
Deimazar et al. (2023) [18]	Adverse drug event (ADE) detection and prediction	Lack of external validation (heterogeneity of EHR data)	Systematic review of 41 studies using NLP for ADE extraction	Few models externally validated; BiLSTM-CRF performed best for ADE detection, but overall quality was low	Iran
Carayon et al. (2017) [19]	Medication error surveillance in the ICU	EHR-related medication errors (information integrity)	Comparison of EHR-related vs. non-EHR-related errors within the same cohort	34% of 1,622 events were EHR-related and had higher potential for patient harm	United States
Tabassum et al. (2024) [20]	Insider‑threat anomaly detection in hospital EHRs	Unlabeled anomalies & high false‑positive rate	Isolation‑Forest‑based models vs. Local Outlier Factor variants	Isolation Forest + SVM reached 99.2% accuracy and 99.8% sensitivity on a North‑England hospital EHR	United Kingdom
Sauer et al. (2022) [21]	Guidance for choosing public ICU datasets	Completeness, richness & heterogeneity across ICU databases	Head‑to‑head SQL analysis of 4 open ICU datasets	Large differences in size, vitals frequency, therapy intensity; choose dataset to fit question	Netherlands
Islam et al. (2023) [22]	Early sepsis prediction from EHRs	Dataset heterogeneity & lack of external validation (proxy for data‑quality differences)	Systematic review contrasting model performance (AUROC) across independent cohorts	Reported AUROC ranged 0.80–0.97 across 42 studies, underscoring performance drops when data definitions differ	Malaysia
Ozonze et al. (2023) [23]	Automated EHR data‑quality assessment	Completeness & value‑conformance checks	Scoping review of 23 automated DQA programs (2011‑2021)	15/23 tools targeted completeness and 12/23 value conformance; efforts remain fragmented and theory‑light	United Kingdom
Ren et al. (2024) [24]	Benchmarking EHR imputation methods	Missing‑data handling under MCAR/MAR/MNAR scenarios	Systematic review comparing 26 statistics vs. ML/deep‑learning methods	ML (Med.KNN, GAN, CA‑TS) usually outperformed MICE; no universally best method	China
Fraser et al. (2024) [25]	HIV care monitoring via EHR alert system	Completeness, accuracy, and timeliness of EHR vs. paper records	Compared 3,467 records with and without automated clinical alerts	Alerts improved data completeness: viral load completeness rose from 11.9% to 26.7%	Rwanda
Lewkowicz et al. (2020) [26]	Economic impact of EHR‑based CDS interventions	No explicit data‑quality issue reporte*d*	Systematic review comparing reported cost outcomes across 27 studies	22/27 studies reported healthcare‑cost reductions, but cost components and methods were highly heterogeneous	Germany
Graber et al. (2019) [27]	Detecting EHR‑related patient‑safety issues in malpractice claims	System‑ and user‑side EHR failures causing information‑integrity problems	Retrospective cohort of 248 malpractice cases coded for sociotechnical factors	Medication (31%) and diagnostic (28%) errors dominated; >80% of events produced moderate or severe harm	United States
Shi (2024) [28]	Causal Dose-Response Estimation/Critical Care Data Analysis/Precision Oncology	Systematic biases and missing data patterns & race, sex, and age	Development, implementation, and evaluation of advanced biostatistical and machine learning methods for analyzing complex biomedical data	HAL-based estimator successfully provides valid and robust inference for causal dose-response curves; ICU data revealed that measurement patterns are strongly biased by patient demographics	United States
Rokade, Mishra (2024) [29]	Illness prognosis and diagnosis from analyzing EHR	Data being uncurated/Inadequate data protection/Vulnerabilities in traditional cloud-based storage systems	Comprehensive literature review, theoretical analysis, and the proposal of a new system architecture and algorithm	Proposal of an architecture and algorithm that integrates machine learning with EHRs on a blockchain network	India
Shooshtari (2021) [30]	Predict patient mortality in the ICU using machine learning models	Missing Data/Class Imbalance/Signal Discontinuities	The study used a 5-hour window for EHR data and a 1-hour window for waveform data to predict mortality over a subsequent 12-hour period	Combining EHR data with biomedical waveforms proved superior for predicting patient mortality, outperforming models that used either data source alone. The top model achieved an AUROC of 0.877.	United States
Ho et al. (2018) [31]	Predict in-ICU mortality	Emulated and investigated types of data quality disparities found across different EMR systems	Performance of three machine learning algorithms: logistic regression (LR), multilayer perceptron (MLP), and recurrent neural network (RNN)	Model generalization to a new ICU led to a ~ 10% AUROC drop. With only 10% of training data, models achieved an AUROC of 0.773, rivaling the PIM2 clinical score of 0.774	United States
Mohamadlou et al. (2018) [32]	Early detection and prediction of Acute Kidney Injury	Missing data and irregularly timed measurements from EHR	ML algorithm’s performance was compared against the Sequential Organ Failure Assessment	A ML algorithm predicted severe AKI 72 h early, outperforming the Sequential Organ Failure Assessment score with a higher AUROC of 0.872 versus 0.815	United States

ABP: Arterial Blood Pressure; ADE: Adverse Drug Event; AKI: Acute Kidney Injury; AUROC: Area Under the Receiver Operating Characteristic Curve; AUC: Area Under the Curve; BiLSTM-CRF: Bidirectional Long Short-Term Memory - Conditional Random Field; CDS: Clinical Decision Support; DQA: Data Quality Assessment; EHR: Electronic Health Record; GAN: Generative Adversarial Network; HAL: Highly Adaptive Lasso, HR: Heart Rate; ICU: Intensive Care Unit; LSTM: Long Short-Term Memory; MAR: Medication Administration Record; MCAR: Missing Completely At Random; MAR: Missing At Random; ML: Machine Learning; MNAR: Missing Not At Random; MICE: Multiple Imputation by Chained Equations; NLP: Natural Language Processing; RNN: Recurrent Neural Network; RR: Respiratory Rate; SpO₂: Oxygen Saturation; SPR: Smart Pump Record; SVM: Support Vector Machine

### Missing data patterns and predictive model performance

Missing data in critical care EHRs is substantial and systematically biased [7]. Analysis of the MIMIC-III database demonstrated that missing data rates exceed 80% for some variables, with the pattern of missingness being highly informative rather than random [8]. Over 40% of predictors selected by feature selection methods were missingness indicators, suggesting that the absence of measurements reflects clinical decision-making rather than random omission [9].

The temporal pattern of missing data proves particularly problematic for predictive modeling [6]. Missing data becomes increasingly informative on ICU days 2 and 3, when clinicians become more selective with laboratory test ordering [9]. This creates non-random missingness patterns that can bias ML models toward overrepresenting certain patient populations or clinical scenarios [8].

Demographic disparities in data collection were consistently documented across studies. The HAL-based estimator provided valid and robust inference for causal dose-response curves, while ICU data revealed systematic demographic disparities in measurement patterns, with White patients receiving more frequent blood pressure and oxygen saturation monitoring than Black and Hispanic patients, creating biases in data availability that can perpetuate healthcare inequities through biased ML predictions [10, 28].

### Temporal data quality issues and model performance

Temporal data quality presents unique challenges in critical care environments [11]. Multiple medical devices use different approaches to time management, creating synchronization challenges that affect the ability to establish causality in clinical settings [3]. Studies measuring temporal accuracy found buffer-related delays of 30 milliseconds between data capture and timestamping, with variable network delays adding further uncertainty [12].

The impact on predictive models is substantial. High-frequency physiological data quality deteriorates significantly throughout ICU stays:

1. Heart rate quality degraded at 75% of average ICU length of stay.
2. Respiratory rate quality degraded at 73% of length of stay.
3. Oxygen saturation quality degraded at 69% of length of stay.
4. Arterial blood pressure quality degraded at 60% of length of stay.

Only 52.7% to 66.7% of ICU stays maintained acceptable vital sign data quality throughout the entire admission, severely limiting the reliability of time-series predictive models [13].

### Data completeness bias and machine learning equity

A proposed architecture integrating machine learning with EHRs on a blockchain network highlights the importance of addressing systematic data quality issues, as evidence reveals significant bias amplification in ML applications [14, 29]. Racial bias in ICU mortality prediction models showed 12% higher false positive rates for minority patients, while sepsis detection models demonstrated systematic underprediction for female patients. These disparities reflect underlying data collection biases that become amplified through ML model training [6]. Socioeconomic bias was particularly pronounced, with models trained on academic medical centers showing poor performance in safety-net hospitals. This suggests that data quality issues interact with healthcare access patterns to create models that may inadvertently worsen health disparities [7]. Integrating EHR data with biomedical waveforms further improved mortality prediction, with the best model reaching an AUROC of 0.877 [30], though generalization to a new ICU led to a ~ 10% AUROC drop; notably, with only 10% of training data, models achieved an AUROC of 0.773, comparable to the PIM2 clinical score of 0.774 [31]. Similarly, a machine learning algorithm predicted severe AKI up to 72 h in advance, surpassing the Sequential Organ Failure Assessment score with an AUROC of 0.872 versus 0.815 [32].

### Multi-modal data integration and clinical decision support

Critical care relies on diverse data sources, and quality issues in any modality cascade through integrated ML systems [15]. Copy-paste prevalence reached 82% in residents’ progress notes and 74% in attending physicians’ notes, creating systematic errors in clinical documentation that affect natural language processing applications [16].

Smart pump integration revealed significant discrepancies between EHR medication administration records and actual device data. Studies comparing smart infusion pump records with EHR medication administration records found 321 MAR discrepancies versus 682 smart pump discrepancies, with vasopressors, narcotics, and total parenteral nutrition showing the highest error rates [17].

### Real-time data quality monitoring impact

The implementation of real-time monitoring systems showed measurable improvements in data quality [18]. EHR-related medication errors comprised 34% of all medication errors in ICUs, with one-third having potential for life-threatening harm [19]. Real-time monitoring systems achieved 99.21% accuracy in anomaly detection when properly implemented, with high sensitivity (99.75%) and specificity (99.32%) [20].

However, the clinical impact of these systems depends heavily on integration with clinical workflows. ICU physicians respond to an average of 187 EHR alerts per patient per day, creating significant alarm fatigue that can undermine the effectiveness of quality monitoring systems [1].

### Institutional variability and model generalizability

Cross-institutional studies revealed dramatic variability in data quality that severely limits model generalizability [21]. Analysis of four major ICU databases showed frequency of vital sign recordings varied dramatically: HiRID recorded 31.7 ± 10.2 heart rate measurements per hour compared to MIMIC-IV’s 1.1 ± 0.4 measurements per hour [21].

This variability has profound implications for ML model deployment. External validation studies consistently showed significant performance degradation, with sepsis detection models dropping from internal AUCs of 0.76–0.83 to external validation AUCs of only 0.63 [22]. The Epic Sepsis Model, deployed in hundreds of hospitals, showed positive predictive values as low as 7.6% in external validation compared to vendor claims [22].

### Detection and mitigation methods

The research identified several effective approaches for detecting and mitigating data quality issues [6].

- Automated Detection Systems: 23 data quality assessment programs have been deployed in real world settings, with 15 focusing on completeness assessment and 12 emphasizing value conformances [23]. Advanced machine learning approaches, particularly Isolation Forest algorithms, achieved 99.21% accuracy in detecting anomalous data patterns [20].
- Imputation Strategies: Multi-level multiple imputation showed superior performance for handling missing data in longitudinal ICU datasets, with 84.6% success rates in imputing missing clinical variables and 35.6% average reduction in missing data across clinical variables [24].
- Real-time Validation: Implementation of real-time data validation systems demonstrated significant improvements in data quality metrics. When viral load alerts were implemented, data completeness improved from 11.9% to 26.7% [25].
- Cost-Effectiveness: Financial analysis revealed substantial returns on investment, with $86,400 net benefit per provider over 5 years for comprehensive EHR data quality initiatives [26]. Cost savings included $16,400 from drug cost optimization, $8,300 from reduced radiology utilization, and $2,400 from laboratory cost reduction [26].

### Real-world implementation challenges

Despite technological advances, implementation challenges remain significant [16]. EHR malpractice claims more than tripled from 2010 to 2018, with primary factors including user error, incorrect information in medical records, pre-populating errors, and system access failures during critical moments [27].

## Discussion

### Clinical implications

The evidence demonstrates that data quality issues in critical care EHRs create cascading effects that compromise multiple levels of healthcare delivery [4]. The finding that over 40% of predictive features are missingness indicators rather than actual clinical measurements fundamentally challenges assumptions about ML model development in healthcare [9]. This suggests that missing data patterns may be more informative than the actual recorded values, requiring a complete reconceptualization of how we approach predictive modeling in critical care [6].

The 34% rate of EHR-related medication errors with one-third having life-threatening potential represents a patient safety crisis that demands immediate attention [19]. The fact that smart pump data revealed more medication discrepancies than EHR records suggests that current clinical documentation systems may be providing false reassurance about medication safety [17].

### Methodological considerations

The systematic degradation of vital sign data quality throughout ICU stays poses fundamental challenges for longitudinal predictive modeling [11]. The finding that only 52.7% to 66.7% of ICU stays maintain acceptable data quality throughout the entire admission suggests that many current ICU ML models may be built on fundamentally flawed assumptions about data availability and quality [13].

The dramatic performance differences between internal and external validation (AUC drops from 0.76 to 0.63 for sepsis models) highlight the generalizability crisis in healthcare AI. This suggests that many published ML models may be systematically overestimating their real-world performance due to data quality issues that are not adequately addressed during development [22].

### Equity and bias implications

The documented racial disparities in vital sign measurement frequencies and 12% higher false positive rates for minority patients in ML models represent a critical intersection of data quality and health equity [33]. These findings suggest that data quality issues may be systematically amplifying existing healthcare disparities rather than addressing them [34].

The socioeconomic bias, associated with substantial data heterogeneity, observed when models trained on academic medical centers perform poorly in safety-net hospitals indicates that data quality issues may be creating a “two-tier” system where AI benefits primarily serve well-resourced healthcare environments [14, 26].

### Future directions

The evidence points toward several critical areas for future development [6]:

- Bias-Aware Model Development: The finding that missing data patterns are highly informative suggests a need for new ML approaches that explicitly model missingness as a feature rather than treating it as a nuisance to be imputed [35].
- Real-time Quality Monitoring: The 99.21% accuracy achieved by advanced anomaly detection systems demonstrates the potential for real-time data quality monitoring, but integration with clinical workflows remains challenging [20].
- Interoperability Standards: The dramatic variability in data quality across institutions highlights the urgent need for standardized data quality frameworks that can ensure consistent performance across different healthcare environments [21].
- Equity-Focused Quality Metrics: The documented disparities in data quality and ML model performance across demographic groups necessitate development of bias-aware quality assessment methods that explicitly monitor for equity implications [33].

Future research should prioritize the development of comprehensive data governance frameworks that integrate automated quality control processes with interdisciplinary collaboration between data scientists and clinical professionals. As emphasized by Lijović and Elbers [36], involving data scientists in ICU data management is essential to establishing rigorous quality control processes, including designing automated tools to detect inconsistencies or missing values and collaborating with clinical staff to create comprehensive data dictionaries. Future efforts must focus on validating model outputs with real-world data, cross-referencing recommendations with clinical observations, and ensuring transparency in model decision-making processes to enhance clinician trust and adoption.

**Fig. 2:**
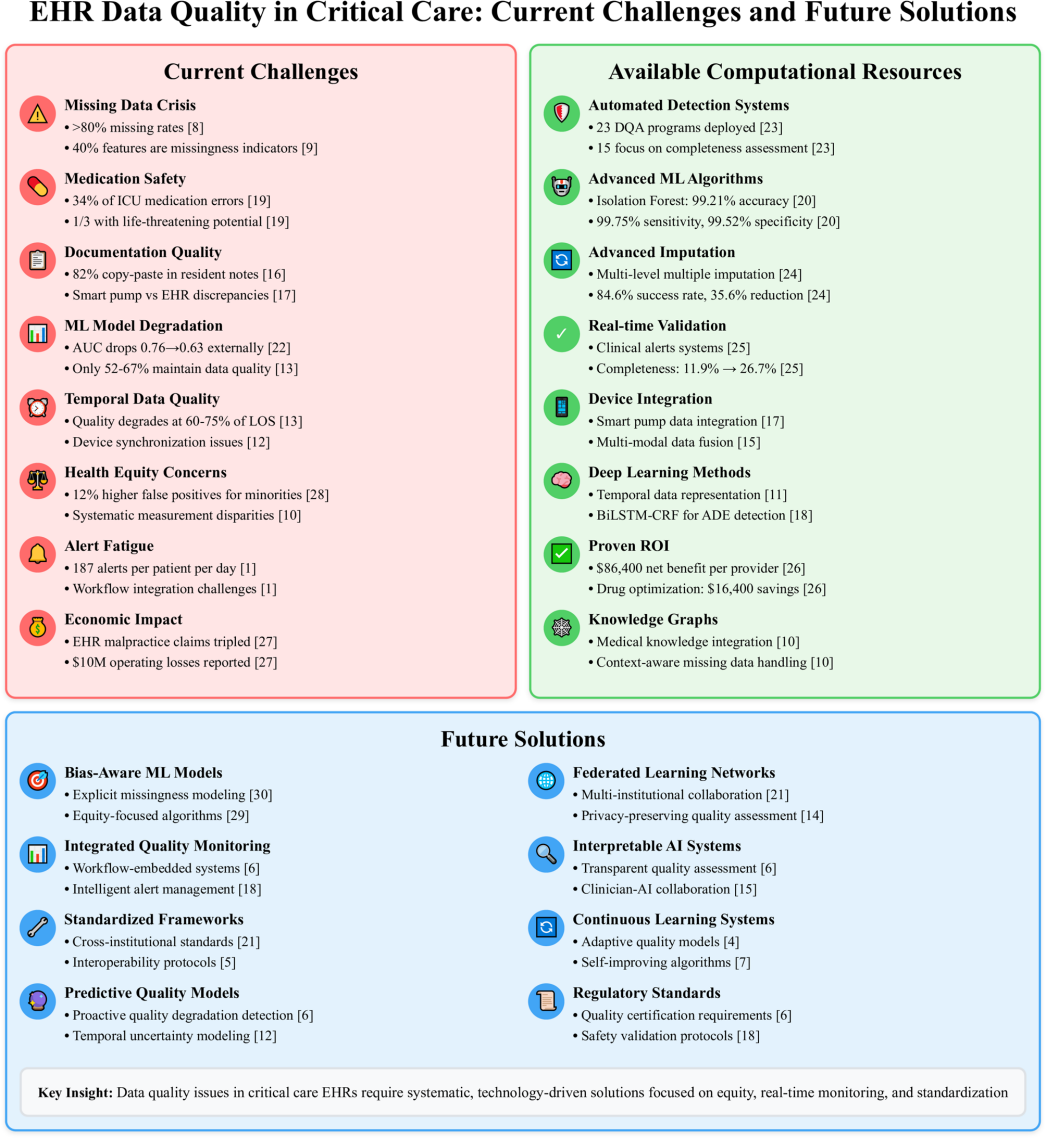
Comprehensive overview of EHR data quality challenges, available computational resources, and future solutions in critical care settings. The three-panel diagram illustrates the current landscape of data quality issues in critical care EHRs (left panel, red), existing computational technologies and resources available to address these challenges (center panel, green), and emerging solutions for future implementation (right panel, blue). Arrows indicate the progressive relationship from problem identification through current technological capabilities to future implementation strategies. ADE, Adverse Drug Event; AUC, Area Under the Curve; BiLSTM-CRF, Bidirectional Long Short-Term Memory - Conditional Random Field; DQA, Data Quality Assessment; EHR, Electronic Health Record; ICU, Intensive Care Unit; LOS, Length of Stay; ML, Machine Learning; ROI, Return on Investment

The Fig. 2 summarizes current evidence, challenges and how the computational resources can address the challenges.

### Open ICU data architectures (MIMIC-IV, eICU-CRD, HiRID, AmsterdamUMCdb, SICdb)

Per the scope defined in our Methods, studies were included only if they measured or mitigated data quality. Foundational papers whose primary contribution is the creation and public release of ICU research databases did not fit those eligibility criteria and were therefore excluded from the quantitative synthesis. Still, these resources shape the downstream landscape, via data model, provenance, de-identification, temporal resolution, and access governance, and thus indirectly condition how data-quality methods behave. For context, we provide a concise architectural overview of five widely used datasets: MIMIC-IV [3], eICU-CRD [37], HiRID [38], AmsterdamUMCdb [39], and SICdb [40, 41], emphasizing system design rather than quality assessment.

MIMIC-IV aggregates EHR data from a single U.S. academic center and introduced a modular schema (e.g., hosp, icu) with a reproducible build pipeline (acquisition → transformation → de-identification with date shifting) under credentialed access, facilitating linkage to companion modules while imposing known constraints on temporal analyses [3]. In contrast, the eICU Collaborative Research Database is multicenter and tele-ICU–sourced, harmonizing heterogeneous site interfaces across >200 hospitals under HIPAA-compliant de-identification and controlled access; by design, its ingestion/standardization layer surfaces between-site heterogeneity that later manifests as cross-hospital variability in completeness and semantics [37].

European datasets add complementary architectural choices. HiRID prioritizes high-time-resolution sampling (most variables every ~ 2 min) from a single center with documented, risk-based de-identification and user training [38]. AmsterdamUMCdb, the first freely accessible European ICU database, couples a risk-based de-identification strategy with governance aligned to HIPAA/GDPR, illustrating privacy-by-design and legal interoperability as first-class system features [39]. SICdb offers minute-level streams with detailed therapy and monitoring; a recent comparative analysis versus MIMIC-IV clarified architectural trade-offs (ICU-only focus and sampling frequency vs. hospital-wide modularity), helping interpret dataset-specific availability and the behavior of downstream quality checks [40, 41]. We do not synthesize these papers quantitatively, but we reference them here to situate the methodological heterogeneity encountered by data-quality studies.

High exclusion reflects our deliberate scope on measuring and mitigating data quality, rather than describing or benchmarking data infrastructures. Foundational “creation” papers primarily focus on system design, governance, and release processes, which, while essential to the ecosystem, do not directly evaluate or propose methods to address data quality issues. As detailed above, these works emphasize architectural aspects, such as modular schema design (MIMIC-IV), cross-hospital harmonization (eICU-CRD), temporal resolution (HiRID, SICdb), or privacy-by-design governance (AmsterdamUMCdb), that shape the downstream behavior of data-quality methods without themselves quantifying or correcting data imperfections. For this reason, such studies were excluded from the quantitative synthesis. Nonetheless, acknowledging their indirect impact, we provide a contextual overview of these architectures to clarify the methodological heterogeneity encountered by subsequent data-quality research.

## **C**onclusions

This systematized review reveals that data quality issues in critical care EHRs are pervasive, systematic, and have profound consequences for ML applications, clinical decision-making, and patient safety. These issues are not merely technical problems but represent fundamental threats to the reliability and equity of healthcare AI systems.

Key findings include missing data patterns that are highly informative rather than random, a high prevalence of EHR-related medication errors, and systematic bias in data collection that amplifies health disparities through ML models. ML model performance also deteriorates significantly during external validation, reflecting broader challenges in generalizability. Although poor data quality imposes substantial financial and operational costs, systematic improvement efforts show clear potential for positive returns.

Critical needs identified include the development of bias-aware ML approaches that explicitly model data quality patterns, implementation of real-time data quality monitoring systems integrated into clinical workflows, standardization of data quality frameworks across institutions, and equity-focused quality assessment methods. The evidence strongly supports systematic investment in data quality improvement as a patient safety imperative. Healthcare organizations must recognize that data quality is not a technical afterthought but a fundamental prerequisite for safe, effective, and equitable AI implementation in critical care.

Future research should focus on comprehensive frameworks that address the intersection of data quality, model performance, and health equity, with critical care serving as a key testing ground for scalable solutions.

## Supplementary Information

Supplementary Material 1

## Data Availability

Not applicable.

## Declarations

### **Guarantor **

Joao Brainer Clares de Andrade
