# Supplementary material for: Discovery of data quality issues in electronic health records: profound consequences for critical care medicine applications – a systematized review
Source: Crit Care. 2026 Jan 8;30:19. doi: 10.1186/s13054-025-05677-0 (PMC12784561; doi:10.1186/s13054-025-05677-0)
Supplement: Supplementary file 1 — Supplementary Material 1 [file 13054_2025_5677_MOESM1_ESM.docx]

**Supplementary material**

**Table 2. Search strategies per database**

| Database | Search strategy |
| --- | --- |
| MEDLINE | ("electronic health record*" OR "EHR" OR "electronic medical record*" OR "EMR") AND ("data quality" OR "data integrity" OR "missing data" OR "data bias*" OR "temporal shift*") AND ("critical care" OR "intensive care" OR "ICU" OR "emergency department" OR "acute care") AND ("machine learning" OR "artificial intelligence" OR "predictive model*" OR "clinical decision support") |
| Embase | ('electronic health record*' OR 'ehr' OR 'electronic medical record*' OR 'emr') AND ('data quality'/exp OR 'data quality' OR 'data integrity'/exp OR 'data integrity' OR 'missing data'/exp OR 'missing data' OR 'data bias*' OR 'temporal shift*') AND ('critical care'/exp OR 'critical care' OR 'intensive care'/exp OR 'intensive care' OR 'icu' OR 'emergency department'/exp OR 'emergency department' OR 'acute care'/exp OR 'acute care') AND ('machine learning'/exp OR 'machine learning' OR 'artificial intelligence'/exp OR 'artificial intelligence' OR 'predictive model*' OR 'clinical decision support'/exp OR 'clinical decision support') |
| IEEE Xplore | ("electronic health record*" OR "EHR" OR "electronic medical record*" OR "EMR") AND ("data quality" OR "data integrity" OR "missing data" OR "data bias*" OR "temporal shift*") AND ("critical care" OR "intensive care" OR "ICU" OR "emergency department" OR "acute care") AND ("machine learning" OR "artificial intelligence" OR "predictive model*" OR "clinical decision support") |
| ACM Digital Library | [[All: "electronic health record*"] OR [All: "ehr"] OR [All: "electronic medical record*"] OR [All: "emr"]] AND [[All: "data quality"] OR [All: "data integrity"] OR [All: "missing data"] OR [All: "data bias*"] OR [All: "temporal shift*"]] AND [[All: "critical care"] OR [All: "intensive care"] OR [All: "icu"] OR [All: "emergency department"] OR [All: "acute care"]] AND [[All: "machine learning"] OR [All: "artificial intelligence"] OR [All: "predictive model*"] OR [All: "clinical decision support"]] |
| CINAHL | ("electronic health record*" OR "EHR" OR "electronic medical record*" OR "EMR") AND ("data quality" OR "data integrity" OR "missing data" OR "data bias*" OR "temporal shift*") AND ("critical care" OR "intensive care" OR "ICU" OR "emergency department" OR "acute care") AND ("machine learning" OR "artificial intelligence" OR "predictive model*" OR "clinical decision support") |
| Web of Science | ("electronic health record*" OR "EHR" OR "electronic medical record*" OR "EMR") AND ("data quality" OR "data integrity" OR "missing data" OR "data bias*" OR "temporal shift*") AND ("critical care" OR "intensive care" OR "ICU" OR "emergency department" OR "acute care") AND ("machine learning" OR "artificial intelligence" OR "predictive model*" OR "clinical decision support") |
| Cochrane Library | ("electronic health record*" OR "EHR" OR "electronic medical record*" OR "EMR") AND ("data quality" OR "data integrity" OR "missing data" OR "data bias*" OR "temporal shift*") AND ("critical care" OR "intensive care" OR "ICU" OR "emergency department" OR "acute care") AND ("machine learning" OR "artificial intelligence" OR "predictive model*" OR "clinical decision support") |
| Google Scholar | intitle:("machine learning" AND "data quality" AND "ICU") AND "electronic health record" |
| DBLP | ("electronic health record*" OR "EHR" OR "electronic medical record*" OR "EMR") AND ("data quality" OR "data integrity" OR "missing data" OR "data bias*" OR "temporal shift*") AND ("critical care" OR "intensive care" OR "ICU" OR "emergency department" OR "acute care") AND ("machine learning" OR "artificial intelligence" OR "predictive model*" OR "clinical decision support") |

Legend. Summary of the search strategies applied to each database for the systematic review.
